# Retinal Ganglion Cells Can Rapidly Change Polarity from Off to On 

**DOI:** 10.1371/journal.pbio.0050065

**Published:** 2007-03-06

**Authors:** Maria Neimark Geffen, Saskia E. J de Vries, Markus Meister

**Affiliations:** 1 Program in Biophysics, Harvard University, Cambridge, Massachusetts, United States of America; 2 Department of Molecular and Cellular Biology, Harvard University, Cambridge, Massachusetts, United States of America; 3 Center for Brain Science, Harvard University, Cambridge, Massachusetts, United States of America; 4 Program in Neuroscience, Harvard University, Cambridge, Massachusetts, United States of America; University of Pennsylvania Medical Center, United States of America

## Abstract

Retinal ganglion cells are commonly classified as On-center or Off-center depending on whether they are excited predominantly by brightening or dimming within the receptive field. Here we report that many ganglion cells in the salamander retina can switch from one response type to the other, depending on stimulus events far from the receptive field. Specifically, a shift of the peripheral image—as produced by a rapid eye movement—causes a brief transition in visual sensitivity from Off-type to On-type for approximately 100 ms. We show that these ganglion cells receive inputs from both On and Off bipolar cells, and the Off inputs are normally dominant. The peripheral shift strongly modulates the strength of these two inputs in opposite directions, facilitating the On pathway and suppressing the Off pathway. Furthermore, we identify certain wide-field amacrine cells that contribute to this modulation. Depolarizing such an amacrine cell affects nearby ganglion cells in the same way as the peripheral image shift, facilitating the On inputs and suppressing the Off inputs. This study illustrates how inhibitory interneurons can rapidly gate the flow of information within a circuit, dramatically altering the behavior of the principal neurons in the course of a computation.

## Author Summary

The eye communicates to the brain all the information needed for vision in the form of electrical pulses, or spikes, on optic nerve fibers. These spikes are produced by retinal ganglion cells, the output neurons of the retina. In a popular view of retinal function, each ganglion cell responds to a small region of interest in the visual image, known as its receptive field, and is specialized for certain image features within that window. When a cell encounters that image feature, the neuron responds by firing one or more spikes. Different neurons are tuned to different features. For example, some ganglion cells fire when light dims, others when it brightens. Here we show that a rapid shift in the image on the retina can cause a dramatic change in a neuron's preferred feature: For example, a dimming-detector can briefly turn into a brightening-detector. We explore the mechanisms that implement such a switch of feature tuning, and the consequences it might have for visual processing.

## Abstract 3

A peripheral image shift produces a transient switch in retinal ganglion cell responses from Off-dominated to On-dominated. This modulation is exerted at least in part presynaptically, presumably at the bipolar cell synaptic terminal.

## Introduction

In the retina, signals flow along parallel, spatially distributed pathways [1]. A major split occurs at the very first synapse: On bipolar cells and Off bipolar cells respond to light with the opposite sign. Retinal ganglion cells receive excitatory input from On or Off bipolars within their dendritic field. Accordingly, On ganglion cells are excited by an increase of illumination in the receptive field center, and Off ganglion cells by a decrease. In addition, certain ganglion cells draw on both bipolar cell types, and are excited transiently both at the onset and the offset of illumination [2,3].

Light falling on the receptive field surround generally antagonizes the action of light in the center: An On cell will be inhibited and an Off cell excited. By contrast, moving stimuli in the receptive field surround can cause a great variety of effects. Pure image motion in the periphery, without stimulation of the receptive field center, is often found to excite retinal ganglion cells [4–7], although certain moving patterns produce inhibition [8–10]. When the receptive field center is stimulated as well, peripheral motion generally suppresses the cell's sensitivity to the center [11–14], with the occasional differing report [6].

In natural vision, large displacements of the entire visual field are very common [15,16]. Some animals, including humans, make eye saccades several times a second, whereas others move their heads to explore a visual scene; in either case, the image shifts rapidly over the retina. Human visual sensitivity decreases dramatically during a saccade, and also during passive image shifts in absence of eye movements [17,18]. Thus at least part of the suppression is triggered by the visual stimulus, rather than central eye control signals. Both saccades and passive peripheral image shifts inhibit visual responses in the lateral geniculate nucleus [18,19], and this may well be the site of the perceptual suppression [20].

The picture emerging from these studies is that each visual neuron monitors events within its receptive field, but the gain of its response to these stimuli can be modulated by global shifts in the image. Here we present evidence from retinal ganglion cells showing that an image shift can have more profound effects by fundamentally altering what stimulus features the neuron will sense. In extreme cases, this leads to the transient conversion of an Off response into an On response. Exploring the retinal circuits underlying this response reversal, we found that the effect of an image shift can be mimicked by depolarizing certain amacrine cells. These are part of the lateral network that communicates global image motion to the ganglion cells.

## Results

We recorded extracellularly from retinal ganglion cells in the isolated salamander retina. To probe how a ganglion cell responds to light, we projected a flickering spot that covered the receptive field center and slightly beyond (Figure 1A). The spot intensity was modulated in a pseudo-random fashion, and the relation between this dynamic flicker stimulus and the neuron's firing rate was fit with a simple linear–nonlinear (LN) model (Figure 1B; see Materials and Methods). The model yields two functions that characterize the neuron's response: the “filter” specifies how light intensity is integrated over time, and the “nonlinearity” accounts for distortions of the response, such as rectification at the bottom and saturation at the top of the firing range [21]. The goal was to test how these basic parameters of the light response were affected by global image shifts. For this purpose, a grating of dark and light bars was added to the stimulus outside the flickering disk. At regular intervals, this grating was shifted rapidly by one bar width (Figure 1A).

**Figure 1:**
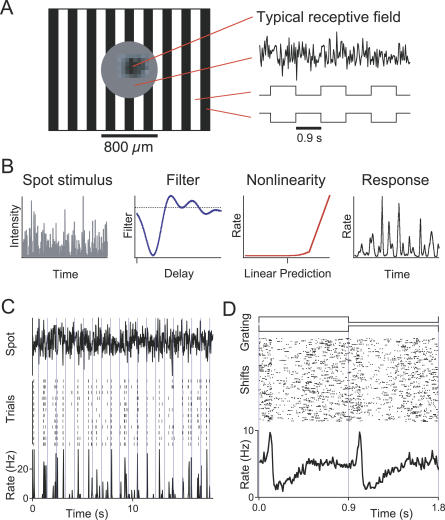
Stimulus, Response, and LN Model (A) Snapshot of the stimulus. A spot covers the center of the display, and a striped grating covers the rest. The receptive field center of a typical ganglion cell is drawn inside the spot. The spot flickers with a pseudo-random intensity (drawn from a Gaussian distribution every 30 ms) while the grating shifts by one bar width every 0.9 s. (B) LN model for the ganglion cell response to the flicker stimulus. The stimulus intensity waveform is convolved with a filter function, and the result is transformed by a static nonlinearity to yield the model's predicted firing rate. The filter and the nonlinearity are adjusted for the best fit to the cell's measured firing rate. (C) Responses of a sample ganglion cell. Top: time course of center spot flicker stimulus. Middle: raster plot of spikes on 12 trials with the identical flicker sequence. Vertical lines indicate times of the peripheral grating shift. Note that the cell's spikes are time-locked to the center stimulus. Bottom: firing rate averaged over the 12 trials. (D) Responses aligned to the grating shifts. Top: time course of grating stimulus, as indicated in (A). Middle: raster plot of spikes for 300 grating shift periods; each period includes two shifts of the grating by one bar width. The center stimulus followed a different random sequence in each shift period, which accounts for the variability in spike timing (see [C]). Bottom: firing rate averaged over 8,221 shift periods.

Many ganglion cells respond to the flickering disk with sharp firing events [22] whose precise times are reliable over stimulus repeats (Figure 1C). The shifts of the grating do not themselves produce such events, but they can strongly modulate the probability of firing. For the sample neuron in Figure 1D, the image shift causes a transient facilitation followed by longer suppression. Note that the first grating shift has precisely the same effect as the second shift, even though the stimulus change has opposite polarity. Clearly the neuron is driven by the flickering disk, but the shifting peripheral grating seems to modulate the response amplitude and possibly other factors.

### A Peripheral Image Shift Causes Some Cells to Change Center Response from Off to On

To learn more about the effect of the peripheral shift on local visual processing, we computed the LN model parameters for each ganglion cell in successive 100-ms intervals after the shift [23]. Figure 2A illustrates results that were typical for On-type responses. The filter function shows a biphasic positive peak, indicating that the cell is excited by a dark-to-light transition and fires about 100 ms later. When the peripheral shift occurs, the cell's firing rate is transiently boosted more than 6-fold. However, the filter function remains unchanged during this time, indicating that the neuron always encodes the same stimulus features, though with varying numbers of spikes. A typical Off-type response is illustrated in Figure 2B. This neuron was excited by light-to-dark transitions. The peripheral shift strongly suppressed the firing of this cell, yet the filter function remained unchanged. These cases conform to the classical notion that peripheral image motion modulates the gain of the center response, but not its selectivity.

**Figure 2:**
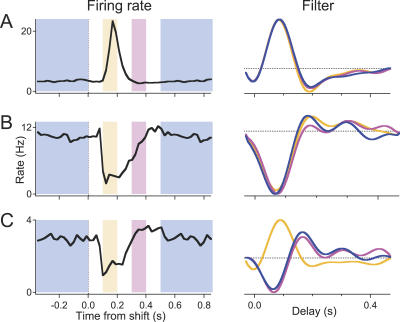
Some Retinal Ganglion Cells Turn from Off-Center to On-Center following a Peripheral Shift Response modulation by the peripheral grating shift is illustrated for three sample ganglion cells: an On cell (A), an Off cell (B), and a polarity-reversing cell (C). Left: changes in firing rate induced by the peripheral shift, displayed as in Figure 1D, bottom. Here and in subsequent figures, the grating shifts of both polarities were averaged. Note that a shift occurred every 0.9 s, so the first and last 0.4 s of these plots are identical. Right: linear filter computed with respect to the flickering spot stimulus in three time periods: the baseline in the 0.4 s preceding the peripheral shift, 0.1–0.2 s after the shift, and 0.3–0.4 s after the shift. The color of the curves corresponds to the time periods on the left.

A very different behavior was found in another group of ganglion cells (Figure 2C). Their filter function had a characteristic Off-type shape, but about 100 ms after the peripheral shift, the filter switched to an On-type shape with a distinct positive peak. This persisted for a short time, and by 400 ms after the peripheral shift, the filter reverted to an Off-type waveform. Thus, the ganglion cell transiently reported visual messages of the opposite sign: increases rather than decreases in intensity. This kind of polarity reversal of the response was observed in 16% of all recorded ganglion cells, but we will show that it is a special case of a much more widespread phenomenon. Polarity reversal was never observed in ganglion cells with On-type responses at baseline.

### Polarity-Reversing Cells Receive On Inputs from On Bipolar Cells

In the following sections, we focus on the mechanism behind polarity reversal in ganglion cells. The shape of the reversed filter of these cells mimics that of a typical On cell (Figure 2), showing that they can receive On-type inputs. This could be via excitation from an On-type bipolar cell (Figure 3A), or inhibition from an Off-type amacrine cell (Figure 3B). To distinguish between the two possibilities, we repeated the experiments in 2-amino-4-phosphono-butyrate (APB), a glutamate receptor agonist that blocks neural transmission to On bipolar cells [3]. As expected, On-ganglion cells produced no light responses in APB, whereas Off ganglion cells continued to respond (unpublished data). Ganglion cells that experienced a polarity reversal by the peripheral shift no longer did so in APB (3/3 cells, Figure 3C): The filter function remained Off-type at all times.

**Figure 3:**
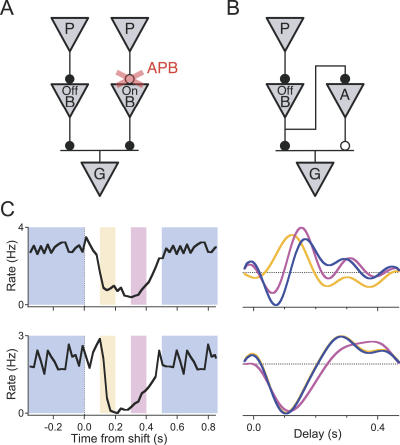
A Polarity-Reversing Ganglion Cell Receives On Inputs from the On Bipolar Pathway (A and B) Two possible circuits underlying On-type responses of ganglion cell, involving photoreceptors (P), bipolar cells (B), amacrine cells (A), and ganglion cells (G), linked by excitatory (closed circles) or inhibitory (open circles) synapses. (A) The ganglion cell is excited by both On-type and Off-type bipolar cells. Application of 2-amino-4-phosphono-butyrate (APB) blocks the On pathway at the photoreceptor synapse. (B) The ganglion cell is excited by Off bipolars, and also inhibited by Off amacrine cells. This On pathway is not affected by APB. (C) Application of APB abolishes polarity reversal. Top: modulation of the firing rate and the linear filter by the peripheral shift, displayed as in Figure 2C for a sample ganglion cell. Bottom: same as top panel, after application of 150 μM APB.

Although APB acts on multiple metabotropic receptors in the retina, the selective loss of On responses is thought to derive from its action on the On bipolar dendrites [24]. Therefore it appears that the polarity-reversing ganglion cells receive direct input from On-type as well as Off-type bipolar cells (Figure 3A). At baseline, the Off-type inputs predominate. The peripheral shift appears to produce a change in the relative weights, such that the On pathway predominates for about 0.1 s.

### A Two-Pathway LN Model

If a ganglion cell indeed receives excitation from both the On and the Off pathways, one expects that some of its spikes are caused by a transient brightening and others by a transient dimming. To test this directly, we inspected the intensity waveforms preceding each of the neuron's spikes, the so-called spike-triggered stimuli. We performed a principal component analysis on this set of waveforms (Figure 4A) and projected them on the two most significant dimensions (Figure 4B). Each spike-triggered stimulus is represented by a point in this space. For the sample neuron of Figure 2C, the points form two clearly separated clusters (Figure 4B). The average waveform of each cluster indeed corresponds to a transient brightening and a transient dimming respectively. Thus, one set of the ganglion cell's spikes can be identified as arising from the On pathway, and the other from the Off pathway. The On cluster disappeared completely when APB was added to the medium (13/13 cells).

**Figure 4:**
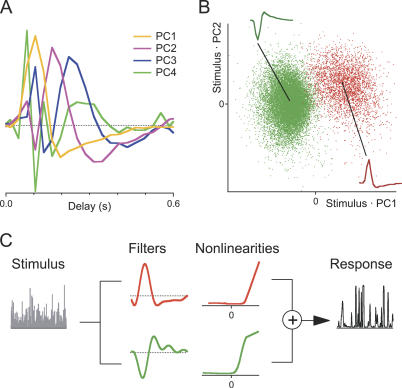
A 2LN Model for Ganglion Cell Responses (A) Principal component analysis (PCA) of the spike-triggered stimulus. For each spike from the neuron of Figure 2C, we identified the stimulus waveform in the preceding 1 s. This set of spike-triggered stimuli was subjected to PCA. The graph shows the first four principal components (PC1–PC4), plotted as a function of time before the spike. (B) The set of spike-triggered stimuli projected onto the first two principal components. Each point derives from one of the cell's spikes. Two clusters are apparent, and the waveforms corresponding to the cluster centers are plotted above or below the respective cluster. (C) Diagram of the 2LN model for this ganglion cell. The stimulus intensity waveform passes through two parallel LN pathways with On and Off filters, respectively. After a rectifying nonlinearity, the outputs of the two pathways are summed to predict the firing rate of the ganglion cell.

Clearly the simple LN model of Figure 1B does not do justice to this confluence of two pathways leading to the ganglion cell. However, a natural extension is to describe each pathway by an LN model and sum their outputs to predict the ganglion cell's firing rate. The filter functions and nonlinearities in each pathway were obtained by fitting separate LN models to the On-spikes and the Off-spikes of Figure 4B. This results in the two-pathway LN (2LN) model of Figure 4C. Note that each of the nonlinearities is strongly rectifying. This means that during a transient brightening in the stimulus, the Off pathway is effectively silent while the On pathway causes firing, and vice versa during a transient dimming. The shapes of the two nonlinearities determine the relative strength of the two pathways. For example, in Figure 4C, the Off pathway has a lower threshold and thus produces more spikes, as is evident from the clusters in Figure 4B.

The mechanism proposed here is a special case of multi-input LN models, and various general methods have been described for estimating the parameters of such models [25–27]. However, in applying the analysis of Figure 4B to 248 ganglion cells, we never encountered more than two clusters, suggesting that only two pathways with distinct nonlinear processing contribute to the response. Thus the 2LN model was found sufficient, and indeed, it performed well in capturing the responses of these ganglion cells: The 2LN prediction for the time course of the firing rate matched the actual time course to better than the trial-to-trial noise in the firing rate (see Materials and Methods).

### The 2LN Model Accounts for Modulation by Peripheral Image Shift

By the above cluster analysis, one can identify which of the ganglion cell's action potentials are caused by the On pathway and which by the Off pathway (Figure 4B). The peripheral shift stimulus left these two clusters intact, but modulated their respective spike numbers (Figure 5A). A raster plot that flags On and Off spikes allows a separate computation of the firing rate caused by the On and Off pathways (Figure 5B), and illustrates the dynamic changes introduced by the peripheral shift. Under baseline conditions just preceding a shift, for example, this neuron is strongly dominated by the Off pathway. About 0.1 s after the peripheral shift, On firing increases sharply whereas Off firing plummets, and the neuron is transiently dominated by the On pathway. We recomputed the 2LN model in successive 0.1-s intervals before and after the shift (Figure 5C). Note that the linear filters obtained for the two pathways remain essentially unchanged in shape at all times. The only change induced by the peripheral shift is in the nonlinearities: The On nonlinearity shifts to the left, transiently lowering the threshold for On signals; whereas the Off nonlinearity shifts to the right. Further, the two nonlinearities have a simple shape: they are monotonically rectifying. By contrast, the best single-pathway LN model (Figure 5C, bottom) requires a time-dependent change of the filter function as well as a change in the nonlinearity, which furthermore takes on a more intricate U-shape. Thus, the move to a 2LN model, even though it introduces additional fitting parameters, actually yields a much simpler description in which the filter functions remain constant in time. This suggests that the peripheral shift does not interfere with the dynamic processing within the On and Off retinal pathways, but merely alters the scalar weighting of their respective outputs at the ganglion cell.

**Figure 5:**
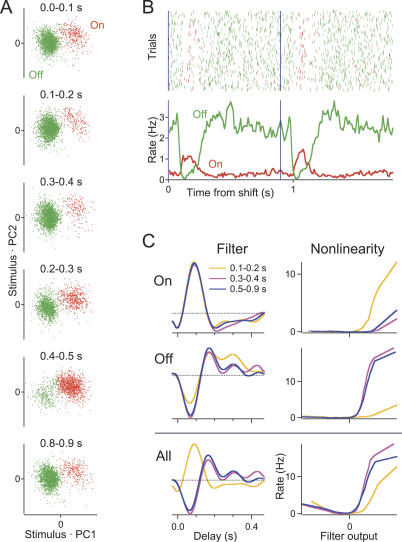
A Peripheral Shift Modulates On and Off Pathways in Opposite Directions (A) The spike-triggered stimuli of a sample ganglion cell, projected onto the first two principal components (PC1 and PC2), as in Figure 4B. Each plot uses spikes from a different time interval relative to the peripheral shift. In all cases, one finds two clusters of stimulus waveforms: one On-type and one Off-type. (B) A raster graph of this cell's firing, displayed as in Figure 1D, but with On spikes and Off spikes indicated separately. Bottom: average firing rate derived from the On and Off pathways. (C) Top: the 2LN model derived from spikes in three different time intervals: 0.1–0.2 s, 0.3–0.4 s, and 0.5–0.9 s. Linear filter and nonlinearity were computed separately from the On spikes and Off spikes as identified by the cluster plot (Figure 5A). Bottom: the single-pathway LN model computed from all spikes. Note the U-shaped nonlinearity to account for both On and Off spikes, as well as the polarity reversal of the filter shortly after the shift.

### A Peripheral Shift Selectively Facilitates On Inputs

The analysis illustrated in Figures 4 and 5 was applied to a sample of 248 ganglion cells, of which 149 cells were driven significantly by both the On and the Off pathway (see Materials and Methods). Among these cells—which we will now call On-Off cells—the relative weighting of the On and Off pathways varied over a wide range of about four orders of magnitude: from 100-fold dominance by Off inputs to 40-fold dominance by On inputs (Figure 6A). Following a peripheral shift, most neurons experienced a transient increase in the On/Off balance. This effect was large: a greater than 2-fold enhancement was seen for 61% of the cells, and a greater than 10-fold change was seen in 19%.

**Figure 6:**
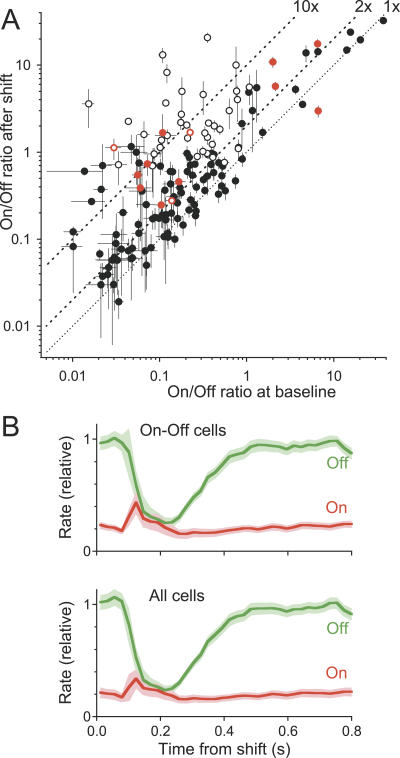
Population Analysis of Peripheral Shift Effects in On-Off Cells (A) Ratio of spikes caused by the On and Off pathways, before and after the peripheral shift. Each point is for one ganglion cell; open symbols represent cells whose linear filter reversed polarity following the shift. Ratios were computed during the 0.1-s period with the largest effect following the image shift (abscissa) and during the baseline period (ordinate, 0.5–0.9 s after the shift). One polarity-reversing cell is not shown because its responses became purely On-type after the peripheral shift. Colored symbols represent neurons in a retina that was subsequently exposed to APB; in APB, all these cells had an On/Off ratio of zero, regardless of the peripheral shift (not shown). Error bars represent one standard deviation uncertainty in the On/Off ratio (see Materials and Methods). (B) Firing rate produced by the On and Off pathways, as a function of time after the peripheral image shift. The firing rate of each cell was normalized to its time-averaged value, and then averaged over the 149 On-Off cells (top) or over all 248 recorded ganglion cells (bottom). Shaded bands report the standard error of the mean.

This transient increase of the On/Off ratio was observed in cells with a wide range of ratios at baseline. A subset of cells (top-left quadrant in Figure 6A) were moderately Off dominated at baseline and switched to moderately On dominated after the image shift. These correspond closely to the cells identified as undergoing polarity reversal by simple visual inspection of the linear filter (Figure 2C). This analysis shows that polarity reversal is merely a special case of a more general phenomenon, namely a relative enhancement of the On pathway, which affects the majority of all retinal ganglion cells in our sample.

Over the whole population of On-Off ganglion cells, the peripheral image shift suppressed the average Off response from 0.1 to 0.4 s (Figure 6B). The On response also showed a slow suppression, but to a lesser extent. In addition, the On response was greatly enhanced over a short period from 0.1 to 0.2 s after the shift. This resulted in a dramatic change in the ratio of On and Off spikes: Whereas this ganglion cell population at baseline mostly emits Off spikes, during the interval from 0.1 to 0.2 s, it produces On and Off spikes in about equal numbers. The same result holds when one extends the analysis over all ganglion cells, including those with pure On and pure Off responses (Figure 6B).

### A Hypothetical Circuit to Explain the Peripheral Shift Effects

Figure 7 depicts a possible neural circuit that accounts for all the observed effects of a peripheral image shift: polarity reversal in ganglion cells (Figure 2C), slow suppression of the Off pathway (Figures 2, 5, and 6B), and transient enhancement of the On pathway (Figures 2, 5, and 6B). The ganglion cell receives inputs from On and Off bipolar cells. The On bipolar input is tonically suppressed by an amacrine cell (labeled A_2_), for example, via inhibition at the presynaptic terminal [28,29], and thus the Off inputs dominate. However, an image shift in the periphery stimulates a second amacrine cell (labeled A_1_), and this neuron in turn suppresses the Off bipolar input through an inhibitory synapse. In addition A_1_ inhibits A_2_ [30], which transiently relieves the suppression of the On bipolar inputs. If the synapse from A_1_ to A_2_ desensitizes rapidly, then disinhibition of the On pathway would be more transient than suppression of the Off pathway, as observed. When both amacrine cells have regained their resting state, the ganglion cell reverts to its baseline off-dominated response.

**Figure 7:**
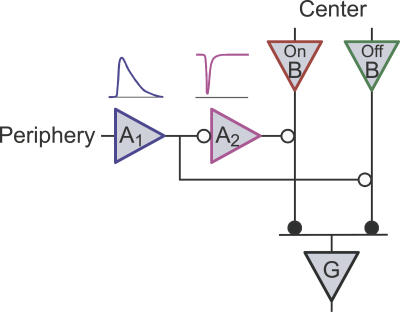
An Amacrine Cell Circuit to Explain Polarity Reversal An On-Off ganglion cell (G) receives excitatory input (closed circles) from On and Off bipolars (B) in the receptive field center, whose gain is modulated by presynaptic inhibition from amacrine cells (open circles). The peripheral shift depolarizes (see time course above A_1_) an amacrine cell (A_1_), which in turn suppresses transmission from the Off bipolar channel. Amacrine A_1_ also inhibits a second amacrine (A_2_), which leads to transient disinhibition (see time course above A_2_) of the On bipolar pathway. See text for details.

### An Amacrine Cell That Facilitates On Inputs and Suppresses Off Inputs to Ganglion Cells

To search for neural elements of the proposed circuit (Figure 7), we recorded intracellularly from many types of amacrine cells [23]. An interesting candidate is illustrated in Figure 8. This is a wide-field amacrine cell (Figure 8A), with processes extending hundreds of micrometers, and thus capable of conveying laterally the effects of a peripheral image shift. Functionally, it is a transient On-type neuron, as revealed by its response to a full-field step of light (Figure 8B). When presented with a shifting grating, this amacrine type responds with a prolonged depolarization lasting about 0.5 s (Figure 8C). The response is identical for shifts in both directions. This can be understood if the neuron pools input from multiple rectified bipolar cells with small receptive fields [31,32]. Note that these properties accord with neuron A_1_ in the circuit of Figure 7.

**Figure 8:**
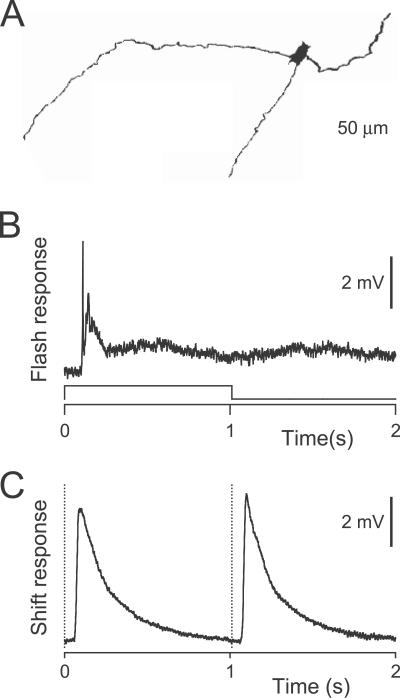
A Peripheral Shift Depolarizes On-Transient Amacrine Cells (A) Intracellular fill of an On-transient amacrine cell in whole-mount view. (B) Intracellular recording of the response to a full-field light flash (bottom trace). Occasionally, action potentials (truncated in this graph) were observed at light onset. Resting potential −77 mV. Single trial. (C) Response to a large grating (see Figure 1A), shifting by a half period every 1 s (vertical lines). Average over 15 trials. Note the depolarizing response is identical to grating shifts of both signs.

To measure the effect of this amacrine cell on the response properties of ganglion cells, we injected 0.5-nA current into the neuron through the electrode while at the same time recording extracellularly from many nearby ganglion cells, and presenting a visual flicker stimulus. The current pulses alternated in sign, and the visual responses of ganglion cells were analyzed separately during periods of amacrine cell depolarization and hyperpolarization. Recall that depolarization simulates the effects of a peripheral image shift on this neuron.

For a typical On-Off ganglion cell, the 2LN model again allowed identification of spikes coming through the On and Off pathways (Figure 9A). Remarkably, depolarization of the amacrine cell modulated the two pathways in opposite directions: The number of On spikes increased by a factor of 1.8, the Off spikes decreased by a factor of 0.75, and thus the On/Off ratio increased by a factor of 2.4 during amacrine cell depolarization (Figure 9B and 9C).

**Figure 9:**
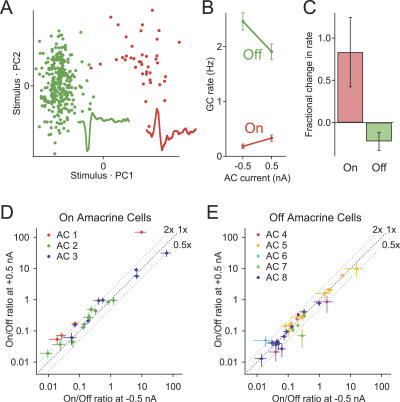
On-Transient Amacrine Cells Control the On/Off Balance of Ganglion Cells An amacrine cell was alternatingly depolarized and hyperpolarized with injection of ±0.5 nA current. Simultaneously, spikes were recorded from ganglion cells under stimulation with a flickering spot (Figure 1A), but no grating. (A) Spike-triggered stimuli for an On-Off ganglion cell, plotted as in Figure 4B, revealing two clusters of On spikes and Off spikes. (B) The firing rate of On spikes and Off spikes, derived from the clusters of (A), during periods of amacrine cell depolarization (+0.5 nA) and hyperpolarization (−0.5 nA). (C) Fractional change in the firing rate of On spikes and Off spikes during depolarization. Note that the On/Off ratio is larger by a factor of 2.4 during depolarization. (D) The effects of stimulating On-transient amacrine cells. For each of three On-transient amacrine cells (AC 1–AC 3), several nearby On-Off ganglion cells were analyzed. For each of these, the graph shows the On/Off ratio during amacrine cell depolarization, plotted against the On/Off ratio during hyperpolarization. (E) The effects of stimulating Off amacrine cells. Two Off-transient and three Off-sustained amacrine cells were stimulated, and the change in On/Off ratio of nearby ganglion cells is plotted as in (D). Error bars in (B–E) represent one standard deviation uncertainty (see Materials and Methods).

We repeated this analysis for three On-transient amacrine cells and all their simultaneously recorded On-Off ganglion cells (Figure 9D). Each of these amacrine cells, when depolarized with 0.5-nA current, caused a substantial increase in the On/Off ratio of nearby ganglion cells. Hyperpolarization produced a comparable decrease in the ratio. The combined effect was a factor of two to three for the most strongly affected ganglion cells. Other ganglion cells remained unaffected. Only one ganglion cell experienced a significant decrease in the On/Off ratio.

For comparison, we repeated the same experiment injecting current into different types of amacrine cells, having Off-sustained or Off-transient light responses (Figure 9E). None of these neurons had a systematic or significant effect on the On/Off ratio of nearby ganglion cells.

These results suggest that a wide-field On-type amacrine cell (Figure 8) contributes to the opposing effects of a peripheral image shift on the On and Off channels in the retina. This amacrine cell has the expected response to a shifting grating and in turn, affects the ganglion cells by suppressing the Off inputs and enhancing the On inputs. Note that our current injection experiment stimulated an individual amacrine cell, whereas a peripheral shift would likely activate a great number of these neurons and thus cause stronger and more reliable effects (Figure 6A).

## Discussion

Large rigid shifts of the retinal image occur every time an animal reorients its gaze, and each such event introduces a new episode of visual processing. The consequences of such image shifts on retinal function have been the subject of great interest. Peripheral motion is commonly found to suppress the sensitivity of ganglion cells to the receptive field center [11–14], but facilitatory actions have been reported as well [6]. The experiments reported here provide a more complete understanding of these shift effects and also give insight into retinal circuitry and the nature of neural coding by ganglion cells. The principal new results are as follows:

First, in the salamander retina, a peripheral image shift transiently suppresses the Off pathway but facilitates the On pathway. Second, in On-Off ganglion cells, this counterphase modulation of two input pathways to the same neuron leads to a dramatic change of response properties: Many cells switch from Off dominated to On dominated for a short period after the image shift. Third, the modulation is exerted at least in part presynaptically, presumably at the bipolar cell synaptic terminal, because different bipolar cell inputs to the same ganglion cell are modulated in opposite directions. Finally, an amacrine cell was identified that mediates the effect of the peripheral image shift on local processing: It has the appropriate visual response and in turn, causes the counterphase modulation of the On and Off pathways.

### Dynamic Neural Coding

A popular view of sensory neurons treats them as “labeled lines”: Each nerve fiber is labeled with a certain sensory feature, and the neuron's firing reports to central brain circuits how prominent that feature is in the stimulus [33]. In this picture, a peripheral image shift leads to a sudden and transient relabeling of optic nerve fibers, and we showed that for some retinal ganglion cells, this relabeling amounts to a complete change in sign of the sensory feature. Under these conditions, the labeled line picture is of limited value, and it becomes necessary to inspect this dynamic process further.

Although all ganglion cell types experience a change in neural coding (Figure 2), the issue is most clearly illustrated by the On-Off cells. Figure 10 plots the visual message of such a neuron for spikes produced at different times before or after the image shift. When there is no motion in the periphery, a spike from this neuron reports a dimming within its receptive field. But shortly after a peripheral image shift, a spike stands for a brightening in the receptive field.

**Figure 10:**
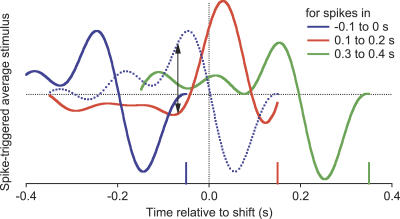
Dynamic Change of a Ganglion Cell's Visual Message The spike-triggered average stimulus of an On-Off cell, plotted for spikes (vertical tick marks) emitted in various 0.1-s intervals surrounding a peripheral shift. Each curve depicts the average intensity time course that caused a spike, and thus represents the average message such a spike sends to the brain. Dotted curve: the visual message that would have been conveyed by spikes in the interval 0.1–0.2 s, if the peripheral shift at 0 s had not occurred. Note that the dotted and solid curves for this interval differ significantly even at time points before the shift (arrows).

One notable aspect of the recoding is its speed: It occurs with a short latency of about 80 ms after the image shift, and the change in sign of the visual message lasts only about 100 ms (Figures 2C, 5B, and 6B). By comparison, the duration of the spike-triggered average stimulus is about 400 ms (Figures 4A and 10). Thus the modulation of the message carried by the neuron occurs on a time scale shorter than the visual message itself. This is in clear distinction to various adaptation phenomena—such as light adaptation [34] and contrast adaptation [23]—for which the coding rules can change on a time scale considerably longer than the message.

As a consequence of this very rapid modulation, one finds that the change in sign of the visual message extends even to time points that precede the peripheral image shift (dashed line in Figure 10). This means that a light stimulus at one point in time may either increase or decrease the neuron's firing, but the decision about this is made only later in time, depending on the occurrence of an image shift. It may seem paradoxical that the report to the brain about one visual event can be altered by a subsequent visual event, but similar effects are well known in human perception, for example in phenomena of “backward masking” [35]. The present instance is easily understood in the context of the circuit model in Figure 7. The temporal integration that determines the 0.4-s time course of the visual message is performed separately in each of the bipolar cell pathways; thus, each bipolar cell holds a record of events 0.4 s in the past. The amacrine cell circuit then simply switches which of those two records gets forwarded to the ganglion cell, without altering the content of the bipolar cell signal (Figure 5C).

### Saccade Processing

In natural vision, large image shifts happen prominently during saccadic eye or head movements [15,16]. What are the functional consequences of the observed “relabeling” for the coding of such visual events? First, it is useful to note the temporal properties of the neural message from the typical ganglion cell. The average stimulus preceding a spike (Figure 10) is a biphasic curve, with almost perfect cancellation between the positive and negative lobes. This means that the neuron reports the difference between the current intensity and the preceding intensity. This is a common feature of ganglion cells in many species [36,37]. Under baseline conditions, long before or after the saccade, the neuron of Figure 10 is excited by a decrease in intensity, but shortly after the saccade, it is excited by an increase in intensity. For spikes emitted during this period, the negative lobe of the visual message falls before the saccade and the positive lobe falls after the saccade. Therefore, these spikes report the change in the retinal image that is introduced by the saccade, whereas spikes occurring earlier or later report changes intrinsic to the visual scene. For saccade-related image changes, this neuron reports a brightening, but for scene-related changes, it reports a dimming. What could be the possible benefit of such a scheme?

Most vertebrate retinas show an asymmetry between the On and Off pathways: Generally the Off ganglion cells are more numerous and have smaller receptive fields [38–40]. Various ecological arguments have been invoked for this; in the case of amphibians, the bias may be related to a behavioral preference for dark hiding spots [15,41]. In the salamander, the predominance of Off-type retinal responses is evident regardless of how one samples the optic nerve signals [42–44]. Even among the On-Off ganglion cells, there is a strong bias toward the Off pathway under baseline conditions [45] (Figure 6A). However, during the brief period that encodes saccade-related changes in the image, this bias transiently disappears, and over the entire population of ganglion cells, the On responses are as frequent as Off responses (Figure 6B). Note that image changes resulting from a rigid shift have an important distinguishing property: comparing all points in the image, the intensity increases are necessarily matched by intensity decreases. This is true even if the image itself is strongly biased, say for small dark objects on a large bright background. Thus, if the goal is to signal the image changes that result from a saccade, it is best to treat brightening and dimming on an equal footing, and devote the same number of On and Off channels. After the saccade, the ganglion cell population reverts to signaling scene-related changes, with an Off bias presumably governed by the ethological goals of visual analysis. The rapid switch allows the retina to use the same resources for two different modes, interleaved in time. Clearly, this interpretation is speculative and would benefit from further insight into the use of saccade-related signals in downstream visual areas.

### Amacrine Cell Circuits

It is a common observation in retinal studies that large-scale image motion causes a suppression of local processing [10–12], and this is thought to be implemented via inhibition from long-range amacrine cells [13,14]. In addition to this suppression, we found a strong transient enhancement that was specific to the On pathway (Figure 6B). We propose that this sign inversion is implemented by a second amacrine cell, which tonically suppresses the On pathway [46], but is itself inhibited by the image shift (Figure 7).

Although alternative circuits can be imagined to implement the observed effects, we identified a type of wide-field amacrine cell that seems to act as proposed in Figure 7. This neuron gets depolarized by a peripheral image shift, and in turn its activity can alter the On/Off balance in nearby ganglion cells by a factor of 2. Because amacrine cells are frequently coupled by electrical junctions, injecting current into one neuron may well depolarize more than one cell. However, the currents used were similar in strength to the synaptic currents under visual stimulation [47], and thus the same events are likely to occur during routine visual processing. This raises the question of how strongly individual amacrine cells in this population are driven by visual stimuli short of a global image shift, for example from local movement within the scene, since such stimuli could result in local modulation of the On/Off ratios among ganglion cells.

Note that these transient On-type amacrine cells (Figure 8) are different from the Off-type polyaxonal amacrine cells involved in the processing of small fixational eye movements [14]. Thus, there is some progress in identifying functions for specific subcircuits and interneurons of the retina, though the intricacy of the inner plexiform layer still leaves many challenges [48]. For example, it is clear that other kinds of amacrine cells also contribute to suppression of the Off pathway by an image shift, since much of this survives when On responses are blocked in APB (Figure 3C).

### Neural Switches

Seen in a broader context, we have illustrated an instance in which two very different signaling pathways are connected to a target neuron, and a neural switch controls which of the pathways is allowed to provide the input signal (Figure 7). Such switching circuits have been postulated previously, to explain dramatic changes in neuronal selectivities. For example, the visual receptive field of a cortical neuron can shift depending on the state of the animal's attention [49]. Also, in experiments that induce binocular rivalry, some cortical neurons appear to be connected alternatingly to the left or the right eye [50]. More generally, the ability to rapidly reroute information flow based on the outcome of an earlier computation is essential in computing machines, and it is difficult to imagine brain function without it [51]. It is intriguing to find the principle used as early as the retina. Here it appears that switching is accomplished by inhibitory local neurons (Figures 8 and 9) that control individual synaptic inputs to the target neuron, presumably through mechanisms of presynaptic inhibition (Figure 7). It will be useful to study this neural switch in more detail, and test whether the mechanisms generalize to other brain circuits.

## Materials and Methods

### Electrophysiology.

To record the spike trains of retinal ganglion cells, the isolated retina of a tiger salamander was placed on a flat array of 61 microelectrodes as described [52] and bathed in oxygenated Ringer's solution at 20 °C–22 °C. Recordings ranged from 2 to 8 h in duration, during which ganglion cells maintained a stable average firing rate. The receptive fields of all ganglion cells were mapped using flickering checkerboard stimuli for about 40 min [52]. We recorded from 81 Off cells, 18 On cells, and 149 cells whose responses were characterized as On-Off, a total of 248 cells in 20 retinas.

To examine the contribution of On bipolar cells, 100 μM APB was added to Ringer's solution for 30–45 min prior to the start of recording.

Sharp electrode recordings from amacrine cells were conducted as described previously [23]. Electrodes were filled with 2 M potassium acetate and 3% Rhodamine Dextran 10,000 MW (Molecular Probes, Eugene, Oregon, United States), with a final impedance of 150–250 MW. After recording, cells were filled iontophoretically (1–3 nA pulses, 5–10 min) and imaged using a confocal microscope with a 40× oil-immersion objective.

### Visual stimulation.

Stimuli were projected onto the retina from a video monitor at a photopic mean intensity of about 10 mW/m^2^. The display (Figure 1A) consisted of a circular spot (800-μm diameter) surrounded by a grating of black and white stripes (width 136 μm or 68 μm). The spot's light intensity was chosen anew every 30 ms from a Gaussian probability distribution with contrast (standard deviation [SD]/mean) of 30%. The surrounding grating shifted by a half period every 0.9 s.

### Analysis.

The relation between ganglion cell firing rate and visual stimulus was fitted by an LN model [21,23] or by a 2LN model. The intensity *s*(*t*) of the circular spot was measured from the video monitor at 1-ms resolution, and normalized to have zero mean, a standard deviation equal to the contrast (0.3), and dimensionless units. In the LN model, this stimulus waveform is first convolved with a linear filter function *F*(*t*), yielding the function

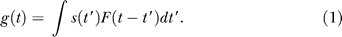



This, in turn, is passed through an instantaneous nonlinear function *N*(*g*) to yield a prediction for the neuron's firing rate

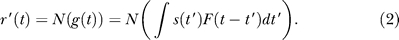



The best-fit filter *F*(*t*) and nonlinearity *N*(*g*) were derived by minimizing the squared deviation between the actual response *r*(*t*) and the prediction *r′*(*t*), as described [23]. Note that the vertical scale of *F*(*t*) trades off with the horizontal scale of *N*(*g*); we resolved the ambiguity by normalizing the filter such that the variance of the filtered stimulus, *g*(*t*), was equal to the variance of the stimulus, *s*(*t*).

In the 2LN model, the predicted firing rate is the sum of two LN pathways,

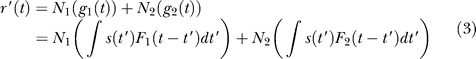



To estimate the two filters, we inspected the set of 1-s stimulus segments *s_i_*(*t*) that preceded each action potential. A principal component analysis of these stimulus waveforms typically yielded four to five significant components (Figure 4A). The stimulus segments *s_i_*(*t*) were then projected onto the subspace spanned by the first two principal components (Figure 4B), although higher dimensions were explored. Generally, this plot revealed two distinct clouds, and the k-Means algorithm was used to sort the points into two clusters. One cluster was found to be centered on an On-type stimulus, the other on an Off-type stimulus, and the spikes belonging to the points were accordingly sorted into On spikes and Off spikes. Then the On spikes and Off spikes were separately fitted by an LN model as described above, yielding the two pathways of the 2LN model (Figure 4C). Note that the principal component subspace served merely to identify the clusters, and all subsequent analysis took place using the full stimulus space.

To evaluate the quality of the 2LN model, we compared the residual between the 2LN fit and the actual firing rate to the trial-to-trial fluctuations in the actual firing rate:

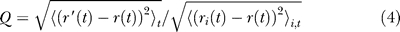
where *r_i_*(*t*) is the firing rate on trial *i,* smoothed with a Gaussian filter of 10 ms SD, *r*(*t*) equals 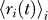 and is the firing rate averaged over trials, *r′*(*t*) is the predicted firing rate, 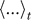 is the average over time, and 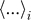 is the average over trials.

Over ten On-Off cells in 12 identical trials, we found *Q* = 0.86 ± 0.08 (standard error of the mean [SEM]). Thus the deviation between the fit and the actual firing rate was smaller than the trial-to-trial fluctuations in the actual firing rate.

For 149/248 ganglion cells, the number of spikes in the smaller cluster was at least 1% of the number of spikes in the larger cluster. We designated these as “On-Off” cells. For such neurons, the firing rate of the On and the Off components was obtained by analyzing separately the spikes in the two clusters (Figures 5B, 6B, 9B, and 9C). The On/Off ratio was calculated as the ratio of the number of spikes in the two clusters observed over a suitable time interval (Figures 6A, 9D, and 9E). All error bars in plots of the firing rate or On/Off ratio represent the one standard deviation uncertainty in that measurement. They were computed assuming that the standard deviation of the number of spikes counted in a cluster is equal to the square root of the spike count. Note that this tends to overestimate the error, because retinal ganglion cells fire with sub-Poisson variability [22].

## Abbreviations

2LN: two-pathway linear–nonlinear
LN: linear–nonlinear

## References

[pbio-0050065-b001] Wässle H (2004). Parallel processing in the mammalian retina. Nat Rev Neurosci.

[pbio-0050065-b002] Miller RF, Dacheux RF (1976). Synaptic organization and ionic basis of on and off channels in mudpuppy retina. III. A model of ganglion cell receptive field organization based on chloride-free experiments. J Gen Physiol.

[pbio-0050065-b003] Slaughter MM, Miller RF (1981). 2-amino-4-phosphonobutyric acid: A new pharmacological tool for retina research. Science.

[pbio-0050065-b004] Barlow HB, Derrington AM, Harris LR, Lennie P (1977). The effects of remote retinal stimulation on the responses of cat retinal ganglion cells. J Physiol.

[pbio-0050065-b005] Fischer B, Krüger J, Droll W (1975). Quantitative aspects of the shift-effect in cat retinal ganglion cells. Brain Res.

[pbio-0050065-b006] McIlwain JT (1964). Receptive fields of optic tract axons and lateral geniculate cells: peripheral extent and barbiturate sensitivity. J Neurophysiol.

[pbio-0050065-b007] Noda H, Adey WR (1974). Retinal ganglion cells of the cat transfer information on saccadic eye movement and quick target motion. Brain Res.

[pbio-0050065-b008] Passaglia CL, Enroth-Cugell C, Troy JB (2001). Effects of remote stimulation on the mean firing rate of cat retinal ganglion cells. J Neurosci.

[pbio-0050065-b009] Demb JB, Haarsma L, Freed MA, Sterling P (1999). Functional circuitry of the retinal ganglion cell's nonlinear receptive field. J Neurosci.

[pbio-0050065-b010] Roska B, Werblin F (2003). Rapid global shifts in natural scenes block spiking in specific ganglion cell types. Nat Neurosci.

[pbio-0050065-b011] Werblin FS, Copenhagen DR (1974). Control of retinal sensitivity. 3. Lateral interactions at the inner plexiform layer. J Gen Physiol.

[pbio-0050065-b012] Enroth-Cugell C, Jakiela HG (1980). Suppression of cat retinal ganglion cell responses by moving patterns. J Physiol.

[pbio-0050065-b013] Cook PB, Lukasiewicz PD, McReynolds JS (1998). Action potentials are required for the lateral transmission of glycinergic transient inhibition in the amphibian retina. J Neurosci.

[pbio-0050065-b014] Ölveczky BP, Baccus SA, Meister M (2003). Segregation of object and background motion in the retina. Nature.

[pbio-0050065-b015] Roth G (1987). Visual behavior in salamanders.

[pbio-0050065-b016] Land MF (1999). Motion and vision: Why animals move their eyes. J Comp Physiol [A].

[pbio-0050065-b017] Mackay DM (1970). Elevation of visual threshold by displacement of retinal image. Nature.

[pbio-0050065-b018] Ross J, Morrone MC, Goldberg ME, Burr DC (2001). Changes in visual perception at the time of saccades. Trends Neurosci.

[pbio-0050065-b019] Noda H (1975). Depression in the excitability of relay cells of lateral geniculate nucleus following saccadic eye movements in the cat. J Physiol.

[pbio-0050065-b020] Thilo KV, Santoro L, Walsh V, Blakemore C (2004). The site of saccadic suppression. Nat Neurosci.

[pbio-0050065-b021] Chichilnisky EJ (2001). A simple white noise analysis of neuronal light responses. Network.

[pbio-0050065-b022] Berry MJ, Warland DK, Meister M (1997). The structure and precision of retinal spike trains. Proc Natl Acad Sci U S A.

[pbio-0050065-b023] Baccus SA, Meister M (2002). Fast and slow contrast adaptation in retinal circuitry. Neuron.

[pbio-0050065-b024] Yang XL (2004). Characterization of receptors for glutamate and GABA in retinal neurons. Prog Neurobiol.

[pbio-0050065-b025] Marmarelis VZ (1997). Modeling methodology for nonlinear physiological systems. Ann Biomed Eng.

[pbio-0050065-b026] Paninski L (2003). Convergence properties of three spike-triggered analysis techniques. Network.

[pbio-0050065-b027] Fairhall AL, Burlingame CA, Narasimhan R, Harris RA, Puchalla JL (2006). Selectivity for multiple stimulus features in retinal ganglion cells. J Neurophysiol.

[pbio-0050065-b028] Tachibana M, Kaneko A (1988). Retinal bipolar cells receive negative feedback input from GABAergic amacrine cells. Vis Neurosci.

[pbio-0050065-b029] Flores-Herr N, Protti DA, Wässle H (2001). Synaptic currents generating the inhibitory surround of ganglion cells in the mammalian retina. J Neurosci.

[pbio-0050065-b030] Zhang J, Wang HH, Yang CY (2004). Synaptic organization of GABAergic amacrine cells in the salamander retina. Vis Neurosci.

[pbio-0050065-b031] Hochstein S, Shapley RM (1976). Linear and non-linear spatial subunits in Y cat retinal ganglion cells. J Physiol.

[pbio-0050065-b032] Demb JB, Zaghloul K, Haarsma L, Sterling P (2001). Bipolar cells contribute to nonlinear spatial summation in the brisk-transient (Y) ganglion cell in mammalian retina. J Neurosci.

[pbio-0050065-b033] Adrian ED (1928). The basis of sensation.

[pbio-0050065-b034] Calvert PD, Govardovskii VI, Arshavsky VY, Makino CL (2002). Two temporal phases of light adaptation in retinal rods. J Gen Physiol.

[pbio-0050065-b035] Breitmeyer BG, Ogmen H (2000). Recent models and findings in visual backward masking: a comparison, review, and update. Percept Psychophys.

[pbio-0050065-b036] Benardete EA, Kaplan E (1999). The dynamics of primate M retinal ganglion cells. Vis Neurosci.

[pbio-0050065-b037] Meister M, Berry MJ (1999). The neural code of the retina. Neuron.

[pbio-0050065-b038] Peichl L, Ott H, Boycott BB (1987). Alpha ganglion cells in mammalian retinae. Proc R Soc Lond B Biol Sci.

[pbio-0050065-b039] Dacey DM, Petersen MR (1992). Dendritic field size and morphology of midget and parasol ganglion cells of the human retina. Proc Natl Acad Sci U S A.

[pbio-0050065-b040] Chichilnisky EJ, Kalmar RS (2002). Functional asymmetries in ON and OFF ganglion cells of primate retina. J Neurosci.

[pbio-0050065-b041] Himstedt W (1967). Experimentelle Analyse der optischen Sinnesleistungen im Beutefangverhalten der einheimischen Urodelen. Zool Jb Physiol.

[pbio-0050065-b042] Vallerga S, Usai C (1986). Relation between light responses and dendritic branching in the salamander ganglion cells. Exp Biol.

[pbio-0050065-b043] Crevier DW, Meister M (1998). Synchronous period-doubling in flicker vision of salamander and man. J Neurophysiol.

[pbio-0050065-b044] Segev R, Puchalla J, Berry MJ (2006). Functional organization of ganglion cells in the salamander retina. J Neurophysiol.

[pbio-0050065-b045] Burkhardt DA, Fahey PK, Sikora M (1998). Responses of ganglion cells to contrast steps in the light-adapted retina of the tiger salamander. Vis Neurosci.

[pbio-0050065-b046] Roska B, Nemeth E, Werblin FS (1998). Response to change is facilitated by a three-neuron disinhibitory pathway in the tiger salamander retina. J Neurosci.

[pbio-0050065-b047] Pang JJ, Gao F, Wu SM (2002). Segregation and integration of visual channels: layer-by-layer computation of ON-OFF signals by amacrine cell dendrites. J Neurosci.

[pbio-0050065-b048] Masland RH (2001). The fundamental plan of the retina. Nat Neurosci.

[pbio-0050065-b049] Moran J, Desimone R (1985). Selective attention gates visual processing in the extrastriate cortex. Science.

[pbio-0050065-b050] Leopold DA, Logothetis NK (1996). Activity changes in early visual cortex reflect monkeys' percepts during binocular rivalry. Nature.

[pbio-0050065-b051] Anderson CH, Van Essen DC (1987). Shifter circuits: A computational strategy for dynamic aspects of visual processing. Proc Natl Acad Sci U S A.

[pbio-0050065-b052] Meister M, Pine J, Baylor DA (1994). Multi-neuronal signals from the retina: acquisition and analysis. J Neurosci Methods.

