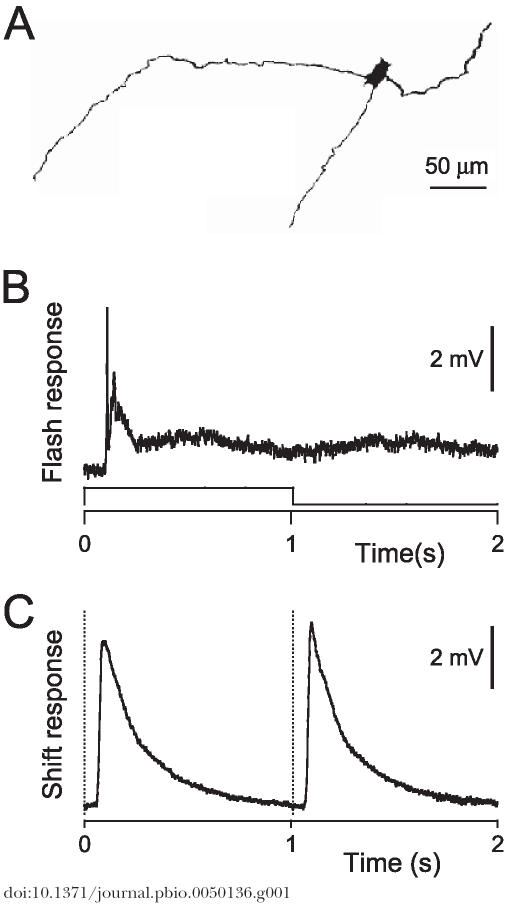# Correction: Retinal Ganglion Cells Can Rapidly Change Polarity from Off to On

**DOI:** 10.1371/journal.pbio.0050136

**Published:** 2007-05-15

**Authors:** Maria Neimark Geffen, Saskia E. J de Vries, Markus Meister

## Abstract

NA

In *PLoS Biology*, volume 5, issue 3: doi: 10.1371/journal.pbio.0050136

Figue 8A was published without a scale bar. The corrected figure is as follows. The legend remains the same.

## 

**Figure:**