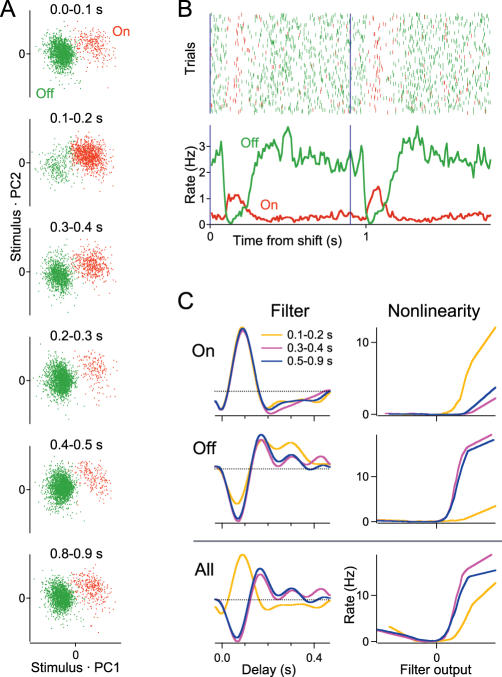# Correction: Retinal Ganglion Cells Can Rapidly Change Polarity from Off to On

**DOI:** 10.1371/journal.pbio.0050188

**Published:** 2007-07-17

**Authors:** Maria Geffen, Saskia de Vries, Markus Meister

In *PLoS Biology*, volume 5, issue 3: doi: 10.1371/journal.pbio.0050065

In Figure 5A, the sequence of the subpanels was inadvertently inverted. The correct Figure 5 is shown here.

## 

**Figure:**